# Supplementary material for: AlbaTraDIS: Comparative analysis of large datasets from parallel transposon mutagenesis experiments
Source: PLoS Comput Biol. 2020 Jul 17;16(7):e1007980. doi: 10.1371/journal.pcbi.1007980 (PMC7390408; doi:10.1371/journal.pcbi.1007980)
Supplement: S1 Table — The overall project accession number is PRJEB29311. (DOCX) [file pcbi.1007980.s001.docx]

Supplementary

**S1 Table**: Conditions evaluated (Triclosan concentrations) and accession numbers for each experiment. The overall project accession number is PRJEB29311.

| **Triclosan (**mg/L) | **Accession number for experiment** | |
| --- | --- | --- |
|  | Replicate 1 | Replicate 2 |
| 0.008 | ERR2854367 | ERR2854368 |
| 0.015 | ERR2854369 | ERR2854370 |
| 0.03 | ERR2854371 | ERR2854372 |
| 0.06 | ERR2854373 | ERR2854374 |
| 0.125 | ERR2854375 | ERR2854376 |
| 0.25 | ERR2854377 | ERR2854378 |
| 0.5 | ERR2854379 | ERR2854380 |
| 1.0 | ERR2854381 | ERR2854382 |
| Control 1 | ERR2854363 | ERR2854364 |
| Control 2 | ERR2854365 | ERR2854366 |
